# Severe Obesity Among Children in New York City Public Elementary and Middle Schools, School Years 2006–07 Through 2010–11

**DOI:** 10.5888/pcd11.130439

**Published:** 2014-07-10

**Authors:** Sophia E. Day, Kevin J. Konty, Maya Leventer-Roberts, Cathy Nonas, Tiffany G. Harris

**Affiliations:** Author Affiliations: Kevin J. Konty, Tiffany G. Harris, Cathy Nonas, New York City Department of Health and Mental Hygiene, New York, New York; Maya Leventer-Roberts, New York City Department of Health and Mental Hygiene, Icahn School of Medicine at Mount Sinai, New York, New York.

## Abstract

**Introduction:**

Although studies have shown that childhood obesity overall is on the decline among New York City (NYC) public school children, the prevalence of severe childhood obesity has not been studied.

**Methods:**

We used height and weight measurements of 947,765 NYC public school students aged 5 to 14 years in kindergarten through 8th grade (K–8), from school years 2006–07 through 2010–11. We used age- and sex-specific body mass index (BMI) percentiles according to Centers for Disease Control and Prevention growth charts to define childhood obesity (BMI ≥ 95th percentile) and severe childhood obesity (BMI ≥120% of 95th percentile) and to identify biologically implausible values (BIV). Multivariable logistic models tested for trends in obesity and severe obesity prevalence. To evaluate misclassification, we recalculated prevalence estimates for the most recent school year (2010–11) including the student records identified as BIV who were also declared severely obese (BMI ≥ 120% of 95th percentile). We refer to this subgroup of BIVs as “high BIV.”

**Results:**

Severe obesity among NYC public school students in grades K–8 decreased 9.5% from the 2006–07 school year (6.3%) to the 2010–11 school year (5.7%), and obesity decreased 5.5% (from 21.9% to 20.7%). The prevalence of severe obesity and obesity was highest among minority, poor, and male children. Severe obesity declined in prevalence among every subgroup, with the greatest effect among white students and wealthy students. Severe obesity prevalence increased with age, and obesity prevalence peaked among those aged 7 to 10 years. For the 2010–11 school year, including high BIVs increased severe obesity prevalence from 5.7% to 6.6% and increased obesity prevalence from 20.7% to 21.5%.

**Conclusion:**

Among all subgroups of NYC public school children in grades K–8, the reduction in severe obesity was greater than the reduction in overall obesity. Efforts to decrease obesity in NYC have affected the severely obese; however, monitoring of this specific subgroup should continue because of differences in trends and greater health risks.

## Introduction

Recent data suggest that the national rise in childhood obesity has started to level off (1,2); however, the rate of severe childhood obesity has more than tripled since the early 1970s and now includes nearly 6% of children in the United States (3–5). Compared with obese children, severely obese children have increased risk for negative health outcomes in adulthood, including cardiovascular disease, insulin resistance, sleep apnea, and severe obesity (6–12). Preventing and reducing the prevalence of severe childhood obesity has serious implications from both a public health and a clinical perspective, which emphasizes the importance of correctly monitoring the severely obese population (3,6,13,14).

Childhood obesity is well documented in New York City (NYC), and an encouraging decrease of 5.5 percentage points among public school children in grades kindergarten through 8th grade (K–8) was seen from the 2006–07 school year to the 2010–11 school year (15). Our main objective was to present the prevalence and trends of severe childhood obesity during this period. A secondary objective was to determine whether the patterns and trends of severe obesity in children differ from those of obesity and to describe the potential shortcomings in applying the Centers for Disease Control and Prevention (CDC) standard biologically implausible value (BIV) definitions in a population health monitoring system to measure and monitor severe childhood obesity.

## Methods

NYC public school students have their height in inches and weight in pounds measured yearly during physical education classes as part of the NYC FITNESSGRAM assessments (16). These data, along with annual enrollment records, were provided to us by the NYC Department of Education for all school years from 2006–07 through 2010–11. We used data from students in grades K–8 aged 5 to 14 years at the end of the school year who were not enrolled in alternative (ie, charter or continuing education) or special education NYC public schools during the 5 schools years studied (15).

By using a unique student identifier, student enrollment records were matched to their FITNESSGRAM results, which included height, weight, birthdate, and measurement date for each year where available. We used SAS 9.2 (SAS Institute, Inc, Cary, North Carolina) and the CDC’s SAS software tool (17) to calculate sex- and age-specific BMI percentiles and to identify outliers, defined as BIVs, by using the World Health Organization’s (WHO) fixed exclusion range (18). Data on students with BIVs were excluded from all analyses with the exception of the 2010–11 school year analysis that explicitly addressed the effects of BIVs (described below). The percentage of enrolled K–8 students with valid BMI measured as part of the NYC FITNESSGRAM program was 60.9% in 2006–07, 76.0% in 2007–08, 85.9% in 2008–09, 91.5% in 2009–10, and 93.0% in 2010–11, and the proportion of measurements identified as BIV decreased from 3.0% in 2006–07 to 2.0% in 2010–11. For each school year, observations were weighted by using 1) a raking process to ensure that students were representative of the enrollment population and 2) the following marginal control totals: race/ethnicity, school borough by district public health office (DPHO) neighborhoods (defined by low-income and disproportionate rates of illness and death), free-meal status, grade, sex, age, and school type (elementary vs middle). Full details on the weighting procedures are provided elsewhere (15).

One major challenge in monitoring severe childhood obesity lies in its definition (19–21). The CDC growth chart’s (22) definition of childhood obesity (BMI at or above the 95th percentile) has been useful at capturing those at highest risk of poor health outcomes; however, given the increase in obesity prevalence, this definition is no longer sufficient. As a response, several groups have used the 99th percentile to define severe obesity (3,7,9,14), despite CDC warnings that indicated this definition was a poor fit to the upper range of the empirical distribution (23). A more accurate approach to establishing a cut-off at 120% of the 95th percentile was proposed in 2009 and has since been widely accepted (6,20). We used these established definitions to classify students as obese (BMI ≥ 95th percentile) and further classified obese students as severely obese (BMI ≥ 120% of 95th percentile).

The demographic variables we examined were sex, age, race/ethnicity, and socioeconomic status (SES). Age at the time of height and weight measurement was grouped into 3 categories, 5 to 6 years, 7 to 10 years, and 11 to 14 years. Race/ethnicity was based on parent report and was grouped into 5 categories: Hispanic, non-Hispanic black, non-Hispanic white, Asian/Pacific Islander, and other (including multiple races). SES was captured by using 2 proxies, whether the student received free meals and school neighborhood’s poverty level. Students from a household with income at or below 130% of the federal poverty threshold (FPT) or who receive government assistance are eligible for free meals and considered to be living in poverty (24). As recommended by Krieger and adapted for NYC, a school neighborhood’s SES was defined by using 2000 Census data as the percentage of residents in the neighborhood whose household income is at or below the FPT as follows: very wealthy (<10%), wealthy (10% to <20%), poor (20% to <30%), and very poor (≥30%) (25,26).

All analyses and prevalence estimates for obesity and severe obesity for the 2006–07 through 2010–11 school years were created in SAS 9.2 by using weighted observations and accounted for clustering by school and student. Differences in obesity and severe obesity prevalence within demographic groups were tested by using the Wald statistic. Multivariable logistic models, described elsewhere (15), were constructed to test for trends in prevalence of severe obesity from the 2006–07 through 2010–11 school years. Models were adjusted for school borough by DPHO; free-meal status; place of birth; language spoken at home; and an interaction of age, sex, and race/ethnicity. To test for differences in trends between the obese and severely obese groups by predicting severe obesity conditional on being obese, models were constructed by using the subset of obese observations with severe obesity as the outcome. Significance was determined at *P* < .05.

Because our analyses focused on the upper tail of the BMI distribution, known problems with the ability of the 2000 growth charts to identify BIVs were a concern (19–21,27). By using school year 2010–11 data, the school year with the lowest percentage of measurements deemed to be BIV (2.0%), we categorized the severely obese records (BMI ≥120% of 95th percentile) identified as BIV into high BIV and all other BIV records as nonhigh BIV. We examined the size and pattern of students identified as high BIV and recalculated the severe obesity estimates assuming all high BIVs were actually valid measurements to determine the maximum effect that misclassified BIVs could have on severe obesity estimates.

The CDC recently updated its SAS software tool (17) that established modified *z* scores as an alternative method to identify BIVs (17). These scores were defined by using the distance between the age- and sex-specific measurements corresponding to a *z* score of 0 and 2 where each measurement is given a modified *z* score expressing the measure’s value relative to this distance. Although the previous BIV criteria remain the standard criteria, CDC recommends using modified *z* scores to establish other cut-offs for BIVs (17). We used this guidance with CDC’s suggested cut-off of 8 for BMI records as an alternative cut-off for high BIV to further evaluate the inclusion of high BIVs on 2010–11 estimates of severe obesity.

## Results

The annual number of enrolled students ranged from 631,409 in school year 2006–07 to 635,257 in school year 2010–11. The demographics of the enrolled population were similar across all years by sex, age, and SES (Table 1). Hispanics comprised approximately 40.3% of the student population throughout the study period, and non-Hispanic blacks decreased from 30.9% in 2006–07 to 27.3% in 2010–11. In all years, the majority of students were enrolled in free-meal programs.

**Table 1:** Demographic Distributions Overall and by Selected Characteristics for the Enrollment Population^a^ Among Public School Children in Kindergarten Through 8th Grade Aged 5 to14 Years in New York City for the 2006–07 through 2010–11 School Years

Select Characteristic	School Year, %				
	2006–07	2007–08	2008–09	2009–10	2010–11
**Total, % (n)**	100.0 (631,409)	100.0 (625,407)	100.0 (627,590)	100.0 (635,361)	100.0 (635,257)
**Sex**					
Female	40.9	49.0	49.0	48.9	48.9
Male	51.0	51.0	51.0	51.1	51.1
**Race/ethnicity**					
Asian/Pacific Islander	14.0	14.4	14.9	15.4	15.7
Hispanic	40.3	40.3	40.3	40.3	40.5
Non-Hispanic black	30.9	30.1	29.2	28.4	27.3
Non-Hispanic white	14.5	14.7	15.0	15.2	15.6
**Age-group**					
5-6 years old	21.0	20.7	20.8	21.8	21.7
7-10 years old	43.3	43.3	43.9	44.2	44.4
11–14 years old	35.6	36.0	35.5	34.1	33.9
**Meal status**					
No free meals	39.3	40.3	42.9	41.7	37.8
Free meals	60.7	59.7	57.1	58.3	62.2
**School neighborhood’s SES^b^**					
Very wealthy (<10%)	14.3	15.6	15.8	15.9	16.0
Wealthy (10 to <20%)	33.3	32.2	32.6	32.7	33.1
Poor (20 to <30%)	21.4	21.2	21.4	21.4	21.5
Very poor (≥30%)	30.9	30.9	30.2	30.0	29.4

Abbreviation: SES, socioeconomic status.

a The valid measurements of body mass index (BMI) were weighted to be representative of the enrollment population for each year by race/ethnicity, school borough by district public health office (DPHO) neighborhood (neighborhoods defined by low-income and disproportionate rates of morbidity and mortality), free-meal status, grade, sex, age, and school type (elementary vs middle). Prevalence estimates of severe obesity and obesity reflect the enrollment population. A student’s recorded BMI was considered biologically implausible if it had at least one measure for height, weight, weight-for-height, or BMI that was identified as biologically implausible by the 2000 CDC growth chart *z* score (17) and the World Health Organization’s fixed exclusion criteria (18).

b Percentage of residents in the school’s postal zip code living below the federal poverty threshold (FPT) as defined by the 2000 US Census: very wealthy (<10% of residents living below FPT), wealthy (10 to <20% below FPT), poor (20% to <30% below FPT), and very poor (≥30% below FPT) (26).

In 2010–11, the prevalence of severe obesity was 5.7% among NYC public school students aged 5–14 years in grades K–8, (Table 2), which represented a 9.5% decrease from a prevalence of 6.3% in 2006–07 (*P* value for trend < .001). For all school years, a larger proportion of boys than girls were severely obese, and a larger proportion of Hispanic or non-Hispanic blacks were severely obese than non-Hispanic whites or Asian/Pacific Islanders. The prevalence of severe obesity was highest among students aged 11 to 14 years followed by students aged 7 to 10 years and 5 to 6 years. For both student free-meal status and school neighborhood’s SES status, the prevalence of severe obesity was greatest among poor students and lowest among wealthy students (6.5% vs 4.4% for free-meal status and 7.2% vs 4.0% for neighborhood status, in 2010–11, Table 2).

**Table 2:** Prevalence^a^ of Severe Obesity^b^ Among Public School Children Aged 5 to 14 Years in Kindergarten Through 8th Grade by School Year and Selected Characteristics, New York City, School Years 2006–07 through 2010–11

Characteristic	School Year, %						Relative Decrease from 2006–07 (%)	Adjusted Test For Trend^c^, *P* Value
	2006–07		2007–08	2008–09	2009–10	2010–11		
**Total**	6.3		6.1	5.9	5.8	5.7	9.5	<.001
**Sex**								
Female	5.5		5.2	5.1	5.0	4.9	10.9	<.001
Male	7.2		6.8	6.6	6.6	6.4	11.1	<.001
**Race/ethnicity**								
Asian/Pacific Islander	2.7		2.4	2.3	2.3	2.3	14.8	<.001
Hispanic	7.8		7.6	7.2	7.4	7.2	7.7	<.001
Non-Hispanic black	6.9		6.7	6.8	6.6	6.5	5.8	.001
Non-Hispanic white	4.5		4.1	3.9	3.9	3.7	17.8	<.001
**Age group, y**								
5–6	4.3		4.2	3.9	3.9	3.7	14.0	<.001
7–10	6.8		6.5	6.2	6.2	6.0	11.8	.04
11–14	7.0		6.6	6.7	6.6	6.5	7.1	<.001
**Meal status**								
No free meals	5.5		5.1	4.9	4.7	4.4	20.0	<.001
Free meals	6.9		6.7	6.6	6.6	6.5	5.8	<.001
**School neighborhood’s SES^d^**								
Very wealthy (<10%)		4.9	4.5	4.2	4.1	4.0	18.4	<.001
Wealthy (10% to 20%)		5.5	5.3	5.2	5.2	5.1	7.3	<.001
Poor (20% to <30%)		6.6	6.2	6.3	5.8	5.8	12.1	.001
Very poor (≥30%)		7.8	7.5	7.2	7.4	7.2	7.7	.084

Abbreviation: SES: Socioeconomic status.

a Prevalence estimates were based on valid body mass index (BMI) (kg/m^2^) measurements weighted to be representative of the enrollment population for each year by race/ethnicity, school borough by district public health office (DPHO) neighborhood (neighborhoods defined by low-income and disproportionate rates of morbidity and mortality), free-meal status, grade, sex, age, and school type (elementary vs middle). Prevalence estimates of severe obesity reflect the enrollment population.

b Severe obesity is defined as having a BMI at or above 120% of the 95^th^ percentile BMI-for-sex-and-age cut-off according to the CDC’s 2000 growth charts (20,22). Students having at least one measure for height, weight, weight-for-height, or BMI that was identified as biologically implausible by the 2000 CDC growth chart *z* score (17) and the World Health Organization’s fixed exclusion criteria (18) were excluded from the measured population.

c To test for trend over school years, a multivariate model was built that included a linear term for trend, along with sex, age, race/ethnicity, school borough by DPHO neighborhoods, free-meal status, place of birth, language spoken at home, and an interaction by age, sex, and race/ethnicity as covariates. Both school and student codes were used as cluster variables.

d Percentage of residents in the school’s postal zip code living below the federal poverty threshold (FPT) as defined by the 2000 US Census: very wealthy (<10% of residents living below FPT), wealthy (10 to <20% below FPT), poor (20% to <30% below FPT), and very poor (≥30% below FPT) (26).

**Figure 1:**
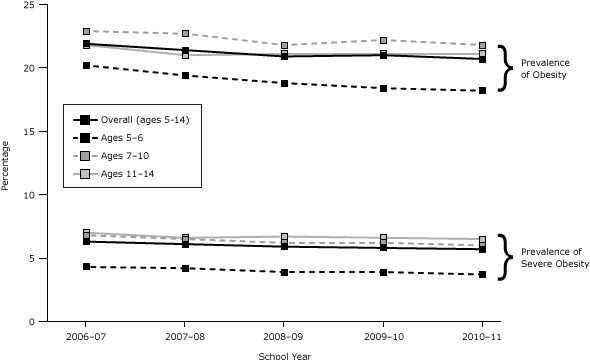
Trends in prevalence by age group of severe obesity and obesity (which includes severe obesity) among NYC public school students aged 5 to 14 years in kindergarten through 8th grade, school years 2006–07 through 2010–11. To test for trend over school years, a multivariate model was built that included as covariates a linear term for trend, along with sex; age; race/ethnicity; school borough by district public health office (DPHO) neighborhoods (neighborhoods with low income and disproportionate rates of morbidity and mortality); free-meal status; place of birth; language spoken at home; and an interaction by age, sex, and race/ethnicity. School and student codes were used as cluster variables. All trends were significant at *P* <.001. Prevalence estimates of obesity (body mass index [BMI] ≥95th percentile) and severe obesity (BMI ≥120% of 95th percentile) are based on valid BMI measurements weighted by race/ethnicity, school borough by DPHO, free-meal status, grade, sex, age, and school type (elementary vs middle) to be representative of the enrollment population for each school year. Adjusted *P* value for trend = .04 for severe obesity among the 7 to 10 years age group. **Table:** 

Severe obesity prevalence decreased among all subgroups from school year 2006–07 to school year 2010–11, with the largest declines in prevalence experienced by subgroups with the lowest prevalence of severe obesity, except by sex, where boys and girls experienced a similar decrease in severe obesity (11.1% vs 10.9%, adjusted *P* value for difference = .08). By race/ethnicity, non-Hispanic whites experienced the greatest decrease (17.8%, adjusted *P* value for trend < .001) followed by Asian/Pacific Islanders (14.8%, adjusted *P* value for trend < .001), Hispanics (7.7%, adjusted *P* value for trend < .001), and non-Hispanic blacks (5.8%, adjusted *P* value for trend = .001). Likewise, wealthier students experienced the greatest decreases in prevalence of severe obesity from school year 2006–07 to school year 2010–11 for both SES measures. By age, the largest prevalence decreases over time (14.0%; adjusted *P* value for trend < .001) were among the youngest age-group (5–6 y), and the smallest decreases (7.1%; adjusted *P* value for trend < .001) were among the oldest age-group (11–14 y) (Table 2) (Figure 1).

Age Group, y	Prevalence	School Year, %					Trend for Time *P* value
		2006–07	2007–08	2008–09	2009–10	2010–11	
Overall (ages 5–14)	Obesity	21.90	21.40	20.90	21.00	20.70	<.001
	Severe obesity	6.30	6.10	5.90	5.80	5.70	<.001
5–6	Obesity	20.20	19.40	18.80	18.40	18.20	<.001
	Severe obesity	4.30	4.20	3.90	3.90	3.70	<.001
7–10	Obesity	22.90	22.70	21.80	22.20	21.80	<.001
	Severe obesity	6.80	6.50	6.20	6.20	6.00	.04
11–14	Obesity	21.80	21.00	21.10	21.10	21.10	<.001
	Severe obesity	7.00	6.60	6.70	6.60	6.50	<.001

The patterns in prevalence and the trends over time of obesity (Table 3) were similar to those of severe obesity by sex, race/ethnicity, and SES and by age group, where trends over time were similar to trends in severe obesity, with modest declines among older aged students (Figure 1). However, the patterns in prevalence of obesity by age group differed from those of severe obesity. For each year, the proportion of obese students that were severely obese increased with each single year of age. This difference is even further emphasized when observing age patterns by race/ethnicity among girls. For example, Hispanic girls peaked in obesity at age 10 (24.2%) and had the lowest obesity prevalence at age 14 (20.0%); however, the proportion of obese students that were severely obese increased from age 10 (25.3%) to age 14 (36.5%) as did the prevalence of severe obesity itself. Other racial/ethnic groups showed a similar pattern with a peak in obesity around age 10, but severe obesity increasing through older ages.

**Table 3:** Prevalence^a^ of Obesity^b^ Among Public School Children Aged 5–14 Years in Kindergarten Through 8th Grade and by School Year and Selected Characteristics, New York City, School Years 2006–07 through 2010–11

Characteristic	Obesity, %						Relative Decrease, 2006–07, %	Adjusted Test For Trend^c^, *P* Value
	2006–07		2007–08	2008–09	2009–10	2010–11		
**Total**	21.9		21.4	20.9	21.0	20.7	5.5	<.001
**Sex**								
Female		19.5	19.1	18.7	18.9	18.6	4.6	<.001
Male		24.2	23.6	23.0	23.1	22.8	5.8	<.001
**Race/ethnicity**								
Asian/Pacific Islander		14.5	13.7	13.2	13.5	13.4	7.6	<.001
Hispanic		26.5	26.0	25.4	25.7	25.6	3.4	<.001
Non-Hispanic black		21.3	21.1	21.2	21.1	20.9	1.9	.015
Non-Hispanic white		17.6	16.9	16.1	16.1	15.4	12.5	<.001
**Age group**								
5–6 years old		20.2	19.4	18.8	18.4	18.2	9.9	<.001
7–10 years old		22.9	22.7	21.8	22.2	21.8	4.8	<.001
11–14 years old		21.8	21.0	21.1	21.1	21.1	3.2	<.001
**Meal status**								
No free meals		20.1	19.4	18.7	18.5	17.6	12.4	<.001
Free meals		23.1	22.7	22.5	22.8	22.6	2.2	.003
**School neighborhood’s SES^d^**								
Very wealthy (<10%)		18.0	17.6	16.7	16.8	16.6	7.8	<.001
Wealthy (10% to 20%)		20.9	20.5	19.9	20.2	20.0	4.3	<.001
Poor (20% to <30%)		22.5	22.2	22.1	21.5	20.9	7.1	.030
Very poor (≥30%)		24.4	23.6	23.4	23.8	23.7	2.9	.660

Abbreviation: SES: Socioeconomic status.

a Prevalence estimates were based on valid body mass index (BMI) (kg/m^2^) measurements weighted to be representative of the enrollment population for each year by race/ethnicity, school borough by district public health office (DPHO) neighborhood (neighborhoods defined by low-income and disproportionate rates of morbidity and mortality), free-meal status, grade, sex, age, and school type (elementary vs middle). Prevalence estimates of obesity reflect the enrollment population.

b Obesity is defined as having a BMI for age and sex at or above the 95th percentile according to the CDC’s 2000 growth charts (22). Students having at least one measure for height, weight, weight-for-height, or BMI that was identified as biologically implausible by the 2000 CDC growth chart *z* score (17) and the World Health Organization’s fixed exclusion criteria (18) were excluded from the measured population.

c To test for trend over school years, a multivariate model was built that included a linear term for trend, along with sex, age, race/ethnicity, school borough by DPHO neighborhoods, free-meal status, place of birth, language spoken at home, and an interaction by age, sex, and race/ethnicity as covariates. Both school and student codes were used as cluster variables.

d Percentage of residents in the school postal zip code living below the federal poverty threshold (FPT) as defined by the 2000 US Census: very wealthy (<10% of residents living below FPT), wealthy (10 to <20% below FPT), poor (20% to <30% below FPT), and very poor (≥30% below FPT) (26).

**Figure 2:**
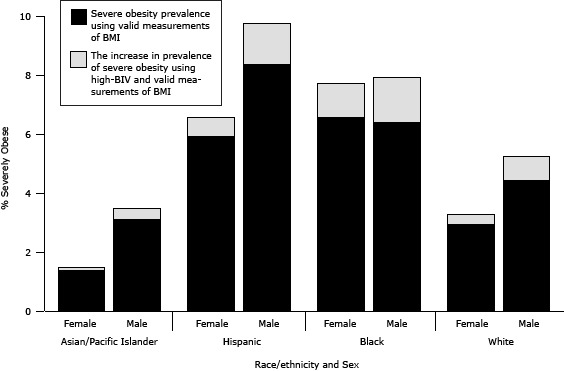
Potential effect of misclassification of plausible values as biologically implausible on severe obesity prevalence, by race/ethnicity and sex among New York City public school students, grades kindergarten through 8, 2010–11 school year. Prevalence estimates of severe obesity (body mass index [BMI] ≥120% of 95th percentile by age and sex) are based on measurements taken during the 2010–11 school year among students aged 5 to 14 years at the end of the school year. Measurements were weighted by race/ethnicity, school borough by district public health office neighborhoods (neighborhoods with low-income and disproportionate rates of morbidity and mortality), free-meal status, grade, sex, age, and school type (elementary vs middle) to be representative of the enrollment population for the 2010–11 school year. Students categorized as Asian/Pacific Islander, black, or white were all non-Hispanic. Persons categorized as Hispanic were of any race. Biologically implausible values (BIV) records are records identified as BIV for height, weight, weight-for-height, or BMI according to the age- and sex-specific Centers for Disease Control and Prevention's 2000 growth chart *z* scores (17) and the World Health Organization’s fixed exclusion range cut-offs (18), which were further classified into high-BIV if the student’s records showed a BMI at or greater than 120% of the 95th percentile. To quantify the upper boundary effect of misclassified BIVs on prevalence estimates of severe obesity, all high-BIV records were assumed to be misclassified as BIV for the 2010–11 school year. The high-BIV measurements were combined with the valid measurements of BMI; these combined records were reweighted (by using the procedures described above) to be representative of the enrollment population for the 2010–11 school year. Prevalence of severe obesity was recalculated including high-BIV records, and the reported percentages are the increase in prevalence observed from including high-BIV records. Abbreviation: BMI, body mass index. **Table:** 

In the 2010–11 school year, 2.0% (n = 12,302) of measurements were classified as BIV and were excluded from prevalence estimates. Of these, 44.7% (n = 5,493) were classified as high BIV (BMI ≥120% of 95th percentile). Of the remaining 55.3% (n = 6,809), which were classified as nonhigh BIV, 91.6% (n = 6,239) were BIV because of an invalid height measurement. Of the nonhigh BIV, 22.3% were underweight (BMI <5th percentile), 31.8% were healthy weight (5th percentile to < 85th percentile), 18.6% were overweight (85th percentile to <95th percentile), and 27.3% were obese but not severely obese (BMI ≥95th percentile to <120% of 95th percentile). Potential misclassification of nonhigh BIV records did not have a meaningful effect on estimates of prevalence, because their distribution among weight classes was approximately proportional. However, 100% of high-BIV records would be classified as severely obese if the measurements were treated as valid. The distribution of high BIVs was similar to the distribution for severely obese by race/ethnicity and sex. If students who were excluded for high BIV were included, the prevalence of severe obesity would increase from 5.7% to 6.6% for 2010–11 and obesity, from 20.7% to 21.5%; however, the increase was not evenly distributed by race/ethnicity and sex (Figure 2).

Race/Ethnicity	Sex	Severe Obesity Prevalence, %	Percentage Point Increase, Severe Obesity Prevalence
Asian/Pacific Islander	Female	1.37	0.10
	Male	3.09	0.40
Hispanic	Female	5.90	0.65
	Male	8.35	1.40
Black	Female	6.55	1.15
	Male	6.38	1.53
White	Female	2.92	0.35
	Male	4.42	0.81

Although not all high-BIV records were incorrectly identified as invalid, recent guidance from the CDC allows us to examine the proportion of these high BIVs that might be valid. By using the BMI modified *z* scores and applying alternative cut-offs suggested in CDC documentation (17), we found that all but 139 of the 5,493 high-BIV measures would now be included as valid for BMI. This suggests that our alternative estimates that include BIVs in the severe obesity calculations may be closer to the true prevalence.

We have not implemented this alternative procedure because this guidance for cut-offs is not fully established, the literature on severe obesity used the previous guidance, and other methods for determining BIVs have been suggested. These include using standard deviations above and below the median values (instead of mean) for the reference population (21,27); by establishing empirical (instead of referenced) age- and sex-specific standard deviations and means to define cut-offs (18); by applying WHO flexible (instead of fixed) exclusion criteria to empirical data (18); and by using longitudinal information (28).

## Discussion

Severe childhood obesity is a subset of childhood obesity that is associated with serious short- and long-term health consequences and is increasing in prevalence among US children (3–6). With its potentially grave consequences, it is important that the subgroup of severely obese children be monitored to inform efforts to reduce the prevalence and prevent incidence of severe obesity (4,6,14). Although it is known that childhood obesity has decreased in NYC (15), the prevalence and trends of severe childhood obesity has not been examined. Ours is the first study to do so.

NYC public elementary and middle school students experienced a decrease of 9.5% in severe obesity from 2006–07 to 2010–11. Among this same population, obesity also decreased during this period by 5.5%, suggesting that the public response to the obesity epidemic is affecting all levels of childhood obesity. We found many similarities in the patterns of prevalence of severe obesity and obesity; both were highest among minority, poor, and male children. However, the prevalence of severe obesity increased with age while the prevalence of obesity peaked in those aged 7 to 10 years and then declined. As age increased, higher proportions of obese students became severely obese. Similar to obesity, severe obesity declined the most among the lowest risk populations (nonminority and wealthy students). We also found that including high BIVs could have a significant effect on prevalence estimates of severe obesity. Our work establishes a baseline for future monitoring of the severely obese pediatric population.

Severe obesity in children is not routinely monitored, despite its conferring the highest risk for poor health outcomes (6). It is important to monitor severe obesity independently of obesity to accurately identify potentially differing trends between the 2 groups. Although we did not find many differing patterns in the prevalence and trends between severe obesity and obesity, the differences by age indicate the potential for other differences that should continue to be monitored. Also, decreases in severe obesity represent an important public health outcome independent of decreases in overall prevalence of obesity. Such decreases may go undetected if we monitor obesity alone, because the severely obese who become obese will continue to be classified as obese. Although the overall goal is to reduce the rate of obesity, a reduction in the rate of severe obesity, even in the context of an increase in overall obesity, has important implications for the health of high-risk populations.

Our data are similar to results from a study of Philadelphia public school children that found obesity rates of 20.5% and severe obesity rates of 7.9% (29). That study also found that groups with the highest rates of severe obesity and obesity at the beginning of the study had the smallest decreases over time. A southern California cohort reported a prevalence of 6.4% among children aged 2 to 19 years enrolled in a prepaid health plan from 2007 through 2008 whose demographic distributions were similar to the southern California census (30). Another study classified children having a BMI at or greater than 120% of the 95th percentile as “Class 2 Obesity” by using National Health and Nutrition Examination Survey data from 1999–2012 for children aged 2 to 19 years. This study reported similar cross-sectional patterns by age, sex, and race/ethnicity with the highest proportions in prevalence found among older (aged 12–19 y), male, and minority children; however, severe obesity prevalence increased from 3.8% in the 1999 through 2000 study period to 5.9% in the 2011 through 2012 period (4).

The CDC’s 2000 growth charts use a standardized distribution derived from populations of children measured from the 1970s through 1990s when 5% of children were declared obese (1,23). These growth charts were not designed to display percentiles beyond 97% (19–21,23,27). The flags for BIV were meant to flag data entry or measurement errors. However, because childhood obesity increased from 5% to nearly 20% from 1980 through 2012 (1), many plausible values are likely for BMI measures that are above the *z* score upper limit defined in the 2000 CDC growth charts. In our analysis, BIVs accounted for 2% of all measurements in the 2010–11 school year. If all high BIVs were found to be plausible, the prevalence of severe obesity among NYC children in grades K–8 would increase by as much as 15%, and the prevalence of obesity would increase by 4%.

Our study has several limitations. The first is that our results do not include private and charter schools, which constitute around 30% of NYC children; these children are not required to participate in NYC FITNESSGRAM. Another limitation is that NYC FITNESSGRAM coverage was low in early years (60.9% in the 2006–07 school year); however, coverage increased each year and was 93.0% in 2010–11. We address this by weighting the measured population to be representative of the enrolled population accounting for individual- and school-level characteristics (15). Although we addressed potential issues with BIV exclusion criteria, which is biased toward lower severe obesity prevalence, it is beyond the scope of this analysis to test new methods of identifying outliers among childhood BMI data. Lastly, the data presented here are cross-sectional. Analyses that examine the BMI trajectories of children with longitudinal records would be able to more accurately determine the true trend of obesity and severe obesity as well as movement into BIVs.

Our study has several strengths. First, the study population was large and diverse (contributing over 2 million BMI measurements), which allowed us to examine severe obesity and obesity by subgroup. Additionally, we examined 5 years of data, which allowed us to examine trends over time. Finally, we were able to show the potential effect of the current BIV definition and to demonstrate the importance of a revised definition that obtains wide acceptance among childhood obesity researchers.

Although obesity is beginning to plateau among US children, severe childhood obesity overall is increasing nationally (4), but not in NYC, as our study showed, where severe obesity is decreasing along with or faster than obesity. Accurately monitoring severely obese populations provides clinicians and public health officials with the ability to target those most at risk for poor health outcomes. Furthermore, given the increased morbidity risks for the severely obese, preventing progression from obesity to severe obesity becomes all the more important (6). Policymakers should make an effort to follow the trends in both severe obesity and obesity while combating the overall problem of childhood obesity. Efforts to decrease overall childhood obesity prevalence in NYC have been successful at decreasing the subpopulation of severely obese children as well.
